# Detection of malaria sporozoites expelled during mosquito sugar feeding

**DOI:** 10.1038/s41598-018-26010-6

**Published:** 2018-05-15

**Authors:** V. A. Brugman, M. Kristan, M. P. Gibbins, F. Angrisano, K. A. Sala, J. T. Dessens, A. M. Blagborough, T. Walker

**Affiliations:** 1 Department of Disease Control, Faculty of Infectious and Tropical Diseases, London School of Hygiene and Tropical Medicine, Keppel Street, London, WC1E 7HT UK; 2 Evolution Biotechnologies, Colworth Science Park, Sharnbrook Bedford, MK44 1LZ UK; 3 Department of Immunology and Infection, Faculty of Infectious and Tropical Diseases, London School of Hygiene and Tropical Medicine, Keppel Street, London, WC1E 7HT UK; 4 Department of Life Sciences, Sir Alexander Fleming Building, Imperial College London, Imperial College Road, South Kensington, London, SW7 2AZ UK; 5 Department of Pathogen Molecular Biology, Faculty of Infectious and Tropical Diseases, London School of Hygiene and Tropical Medicine, Keppel Street, London, WC1E 7HT UK

## Abstract

Malaria is a severe disease of global importance transmitted by mosquitoes of the genus *Anopheles*. The ability to rapidly detect the presence of infectious mosquitoes able to transmit malaria is of vital importance for surveillance, control and elimination efforts. Current methods principally rely on large-scale mosquito collections followed by labour-intensive salivary gland dissections or enzyme-linked immunosorbent (ELISA) methods to detect sporozoites. Using forced salivation, we demonstrate here that *Anopheles* mosquitoes infected with *Plasmodium* expel sporozoites during sugar feeding. Expelled sporozoites can be detected on two sugar-soaked substrates, cotton wool and Whatman FTA cards, and sporozoite DNA is detectable using real-time PCR. These results demonstrate a simple and rapid methodology for detecting the presence of infectious mosquitoes with sporozoites and highlight potential laboratory applications for investigating mosquito-malaria interactions. Our results indicate that FTA cards could be used as a simple, effective and economical tool in enhancing field surveillance activities for malaria.

## Introduction

Malaria is a vector-borne disease caused by parasites of the genus *Plasmodium* and transmitted by Anopheline mosquitoes. *Plasmodium* species affecting humans are responsible for significant mortality and morbidity in tropical countries, with an estimated 216 million cases and 445,000 deaths recorded globally in 2016^1^. The large-scale use of interventions such as long-lasting insecticidal nets (LLINs) and indoor residual spraying (IRS) has demonstrated the benefits of significant transmission reductions^2^. However, the increase in insecticide resistance in mosquito populations is likely to impact the long-term efficacy of these strategies^3,4^. Furthermore, there remains a need to develop novel, effective tools that can be integrated into surveillance and control strategies, especially with the end goal of disease elimination.

The risk of malaria infection is partly dependent on the proportion of mosquito vectors that contain the infectious sporozoite stages of *Plasmodium* parasites. Sporozoites can only be found in female mosquitoes which have lived long enough for the parasites to complete their sporogonic development and migrate from oocysts on the midgut wall to the salivary glands for onward transmission. When an infectious female bites a human host and starts probing, sporozoites are injected with saliva before ingestion of blood. During feeding on a sugar source, saliva is mixed with sugar, initiating digestion which continues in the crop^5^.

Transmission intensity is often represented by the entomological inoculation rate (EIR), which can be mathematically determined by measurement of its component factors, including mosquito density, biting rates, and the sporozoite rate in mosquitoes. Transmission-related metrics such as EIR can be used to assess whether control interventions are having an impact on transmission intensity. However, there are no standard protocols on how to measure EIR components, methodologies used are variable and largely subjective, and a range of other factors affect EIR components potentially increasing error rates^6,7^.

Determining the proportion of wild *Anopheles* mosquitoes with sporozoites has traditionally relied on time-consuming and labour-intensive dissections and microscopical examination of salivary glands^8^ or enzyme-linked immunosorbent assays (ELISA) targeting the circumsporozoite protein (CSP) in homogenates of mosquito heads and thoraces^9^. Pre-sorting is required for morphological identification of *Anopheles* vector species in these studies and determining sporozoite rates in large samples is a demanding process, especially when considering that infection rates can be low even in areas of high transmission. Detection of sporozoites using PCR-based methods has resulted in greater sensitivity, specificity and shorter processing time^10^. The ability to accurately detect *Plasmodium* parasites in *Anophele*s mosquitoes and assess sporozoite rates can have significant consequences on the estimations of malaria transmission intensity, and will be especially important if malaria elimination efforts intensify and malaria case incidence becomes increasingly low^11–13^. New methods will be needed to complement currently used entomological procedures for detection of infectious mosquitoes, as mosquito densities and infection rates decline and secondary vectors become increasingly important^12,14^.

Rodent malaria (*P*. *berghei*) and human malaria (*P*. *falciparum*) sporozoites have been successfully detected in the saliva of infected *Anopheles* females using immunoassays and imaging techniques^15–18^. These studies showed that sporozoites are expelled in saliva without uptake of mammalian blood, for example during sugar feeding. Therefore, it could be possible to exploit the sugar feeding behaviour of adult female mosquitoes to detect malaria sporozoites in the wild, minimizing the need for labour-intensive collection methods or special handling of mosquitoes and preserving them for further studies. Other mosquito-borne disease surveillance studies have developed methods for detection of arthropod-borne viruses (arboviruses) in honey-soaked Flinders Technology Associates (FTA) cards in modified mosquito traps^19^. Mosquitoes expectorated viruses in their saliva while feeding on the FTA cards and laboratory processing using PCR was successfully undertaken to identify viruses. FTA cards preserve RNA and DNA without the need for a cold storage chain which is often problematic in most tropical field settings.

In this study, we show that malaria-infected mosquitoes expel sporozoites during sugar feeding under laboratory conditions by first undertaking forced salivation. We also demonstrate that sporozoites can be detected from sugar-soaked cotton wool (the standard sugar source substrate for maintaining mosquito colonies) and from sugar-soaked FTA cards by real-time PCR. Finally, we provide evidence that *Anopheles* mosquitoes feeding on FTA cards results in significant mortality providing a potential added benefit to using FTA cards for malaria sentinel site surveillance in field settings.

## Results

### *Plasmodium berghei* sporozoite qPCR detection threshold

We first undertook preliminary experiments to ensure that we could accurately detect *Plasmodium* sporozoites using real-time (q)PCR targeting *P*. *berghei cytb*. We dissected salivary glands from 10 *P*. *berghei*-infected *Anopheles stephensi* (SK strain) females at 22 days post infection (dpi) and estimated the total amount of sporozoites that would be present for genomic (g)DNA extraction by counting using a haemocytometer. Initial counts estimated the presence of 58,500 sporozoites, or 5850 per mosquito. Serial ten-fold dilutions until 1:100,000 of DNA extracted from the pooled salivary glands were used to determine the cycle threshold (Ct) value for detection. The serial dilution series of *P*. *berghei*-infected dissected salivary gland extracts resulted in an r^2^ value of 1.0 and slope of −3.21. To further ensure the correct target sequences were amplified, dissociation curve analysis resulted in all extracts producing a single peak of 74.71 +/− 0.08 °C. Using a combination of fluorescence and dissociation curve analysis, we identified a Ct of 34 cycles beyond which we were unable to detect *P*. *berghei cytb*. This was estimated to represent a single sporozoite based on the estimated total present for DNA extraction.

### Forced salivation

**Figure 1:**
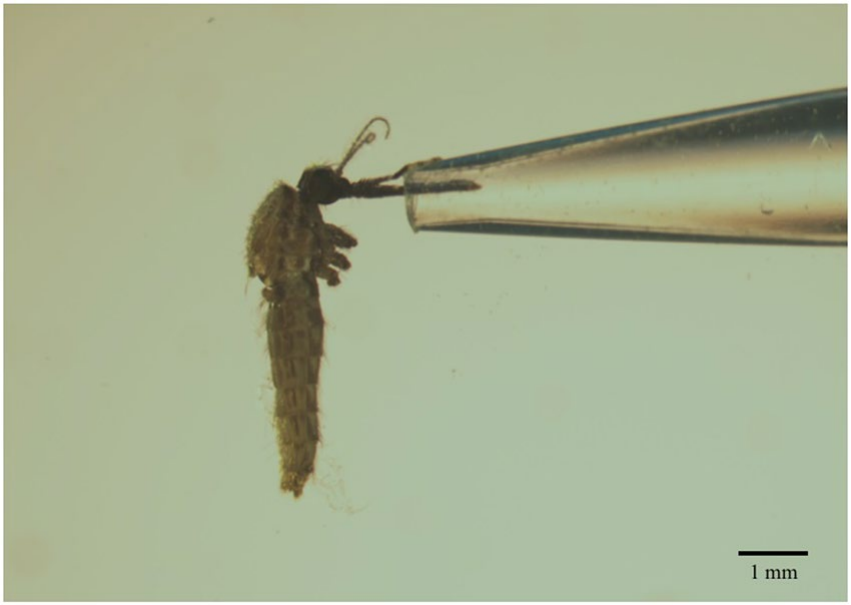
Collection of mosquito saliva (‘forced salivation’). An *An*. *stephensi* female mosquito with legs and wings removed and proboscis inserted into a pipette tip containing 100 μl of 10% glucose solution. Saliva was collected in this manner for two hours per mosquito.

**Figure 2:**
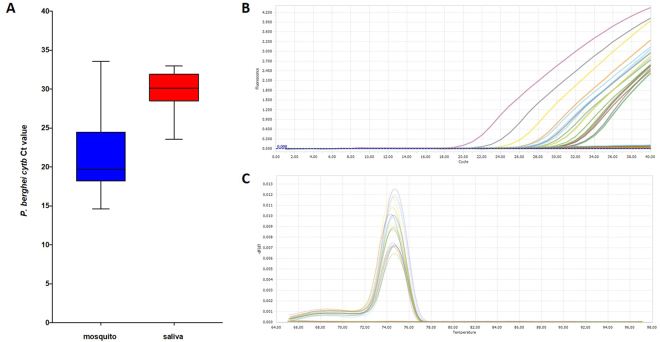
Detection of expelled sporozoites using qPCR. (**A**) Box and whisker plot of qPCR Ct values for the *P*. *berghei cytb* gene in infected adult mosquitoes (blue) and the resulting saliva extracts from forced salivation (red); (**B**) Fluorescent profiles for detection of the *cytb* gene in saliva; (**C**) Dissociation curve analysis indicating correct target amplification.

*Anopheles stephensi* mosquitoes infected with *P*. *berghei* (n = 41) had their proboscis inserted into a 10% glucose solution at 19 days post infection (dpi) and saliva was collected for 2 hours (Fig. 1). Sporozoites were detected in all whole mosquito extracts (41/41) and 19/41 (46.34%) of saliva extracts using qPCR targeting the *P*. *berghei cytb* gene. A mean *cytb* Ct value for mosquitoes of 21.52 +/− 0.73 (median 19.67, interquartile range (IQR) 18.2–24.48) (Fig. 2A) confirmed that significant malaria infection levels were present in all mosquitoes, given that Ct values are inversely correlated to the amount of DNA amplified. A mean Ct value of 29.93 +/− 0.59 (median 30.11, IQR 28.46–31.92) was seen for saliva extracts that produced detectable levels of the *P*. *berghei cytb* gene target (n = 19) (Fig. 2A). Fluorescence detection from saliva extracts were within the 34 Ct cycle threshold (Fig. 2B) and dissociation curve analysis (Fig. 2C) indicated correct amplification of the target. These results using a ‘forced salivation’ method confirm previous studies showing that malaria sporozoites are expelled during sugar feeding^15–17^ and our study demonstrates the ability to use qPCR to detect significant levels of sporozoites in mosquito saliva. It was not unexpected that not all salivary extracts were positive; evidence indicates that sporozoites may not successfully reach the salivary glands in every infected mosquito^20^, and even heavily infected mosquitoes may not eject sporozoites during every feed^21^.

### Detection of *P*. *falciparum* sporozoites in sugar-soaked cotton wool

**Figure 3:**
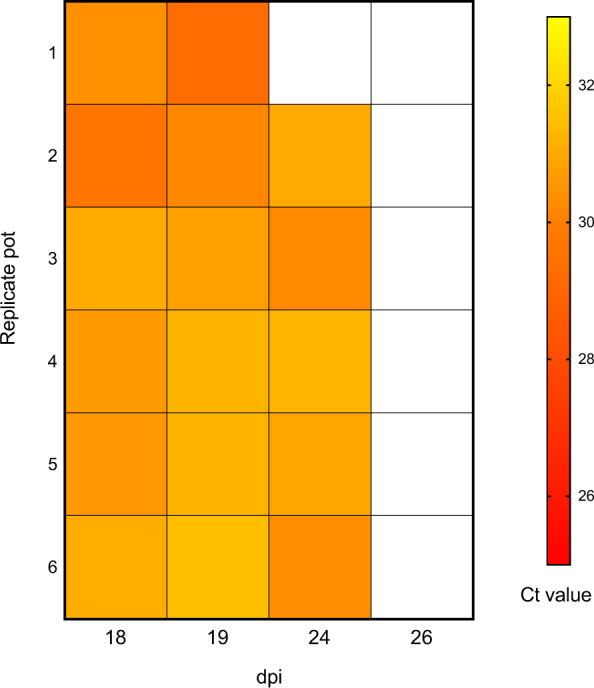
Heat map showing the relative amounts of *P*. *falciparum* malaria sporozoites in saliva. qPCR targeting the *P*. *falciparum cox1* gene^24^ was used to determine the relative amounts in gDNA extracts from saliva in cotton wool collected from 6 replicate pot cages containing ~70 mosquitoes per pot. Lower Ct values (red) indicate a higher level of sporozoite DNA detected and white indicates absence of detection.

We then went on to determine if we could detect malaria sporozoites under more natural feeding conditions in the laboratory using human malaria (*P*. *falciparum*)-infected *An*. *stephensi* mosquitoes. Infected mosquitoes in 6 replicate groups of ~70 were provided with a sugar source (10% glucose) via cotton wool pads, which were changed every 24 hours. DNA was extracted from cotton wool pads removed at days 18, 19, 24 & 26 dpi, using a modified protocol (see methods section). qPCR analysis targeting the *cox1 P*. *falciparum* gene^22^ was undertaken on cotton wool pad extractions and we sub-sampled mosquitoes from each pot (n = 10) to estimate the infection prevalence after the feeding regime had finished. As shown in Fig. 3, we could detect *P*. *falciparum* sporozoites in cotton wool samples from 18, 19 & 24 dpi but not from 26 dpi. The mean infection rate of mosquitoes in the replicate groups was 56.67 +/− 7.60 with a mean Ct for *P*. *falciparum cox1* for the infected individuals (n = 34) of 28.31 +/− 0.33. These results suggest the possibility that when sporozoite-infected mosquitoes are presented with repeated sugar feeding opportunities the sporozoite levels may decrease due to continued expulsion of saliva.

### Detection of malaria sporozoites in FTA cards

As FTA cards represent the optimal substrate to preserve DNA in field settings, we compared our ability to detect *P*. *berghei* sporozoites from Whatman indicating FTA cards (Whatman International Ltd, Maidstone, Kent, UK) with the standard cotton wool setup. Mosquitoes infected with *P*. *berghei* were divided into replicate groups of 5 and 10 at 22 and 23 dpi, with excess mosquitoes (~20) maintained in a standard infection cage. Mosquitoes were presented with the opportunity to feed on either FTA cards or cotton wool (both soaked in 10% glucose solution) for a 24-hour period. DNA was extracted from FTA cards, cotton wool samples and mosquitoes in replicate groups. For the main infection cage, both an FTA card and a cotton wool pad were simultaneously provided at 22 dpi for 24 hours to determine if sporozoites could be detected from both substrates (Supplementary Figure S1A).

**Figure 4:**
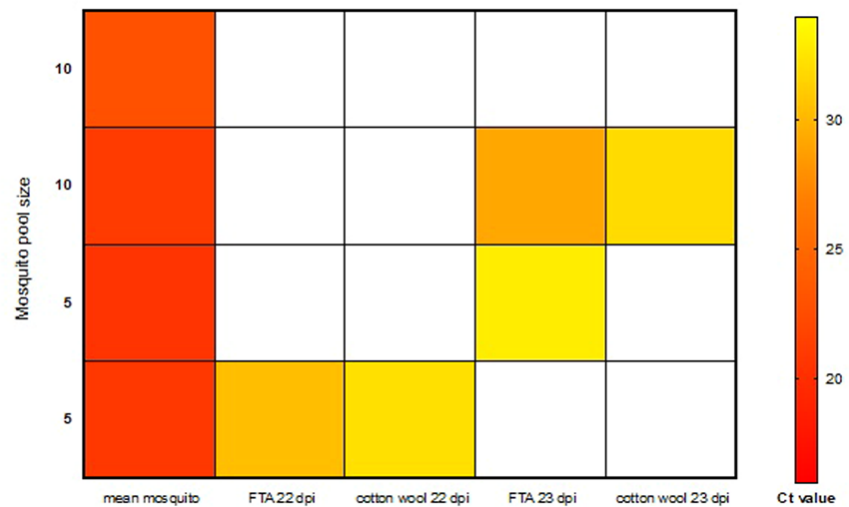
Heat map showing the relative amounts of mean *P*. *berghei* malaria parasites in mosquitoes and sporozoites in saliva eluted from FTA cards and cotton wool. Sugar substrates were placed on pots of mosquito pools of 5 and 10 with two replicate pots for each pool size over two days of collection. qPCR targeting the *P*. *berghei cytb* gene was used to determine the relative amounts and lower Ct values (red) indicate a higher level of sporozoite DNA detected and white indicates absence of detection.

*Plasmodium berghei cytb* PCR analysis on DNA extracts from the infection cage revealed Ct values of 29.3 and 31.9 for the FTA card and cotton wool pad respectively. This demonstrates that mosquitoes can feed on and expel sporozoites onto FTA cards with a potentially greater sensitivity of detection observed when compared to cotton wool. For mosquitoes in replicate groups of 5 and 10, we detected sporozoites from both FTA cards and cotton wool at both 22 and 23 dpi (Fig. 4), further confirming that FTA cards provide a suitable substrate from which to detect malaria sporozoites. All mosquitoes used in groups of 5 and 10 were subsequently shown to be positive for *P*. *berghei* sporozoites at 24 dpi with a mean Ct value of 21.32 +/− 0.55. All our Ct values from FTA cards and cotton wool were within the threshold limit of detection (34 cycles) and had identical dissociation curves with single peaks.

We repeated the experiment using 24 replicate groups containing three *P*. *berghei*-infected mosquitoes (Supplementary Figure S1C). Half of the groups were provided the opportunity to feed on FTA cards, the other half on cotton wool at 21 and 24 dpi. As shown in Table 1, at 21 dpi 36% of FTA card DNA extracts were positive for *P*. *berghei* sporozoites compared to 31% of cotton wool DNA extracts. A similar trend was observed at 24 dpi but greater detection was observed, with 58% of FTA cards and 55% of cotton wool DNA extracts having detectable levels of *P*. *berghei* sporozoites. Mean Ct values were comparable across sugar substrates and dpi suggesting FTA cards provide a more consistent substrate for both preservation of DNA and elution during the DNA extraction process.

**Table 1:** *Plasmodium berghei cytb* PCR analysis on DNA extracted from FTA cards and cotton wool from 24 replicate groups of 3 mosquitoes at 21 & 24 days post infection (dpi).

dpi	FTA cards		Cotton wool	
	% detection	mean Ct value	% detection	mean Ct value
21	36.4	29.92 +/− 1.30	30.8	30.65 +/− 1.09
24	58.3	31.24 +/− 0.64	54.6	32.35 +/− 0.59

### Mosquito survival

**Figure 5:**
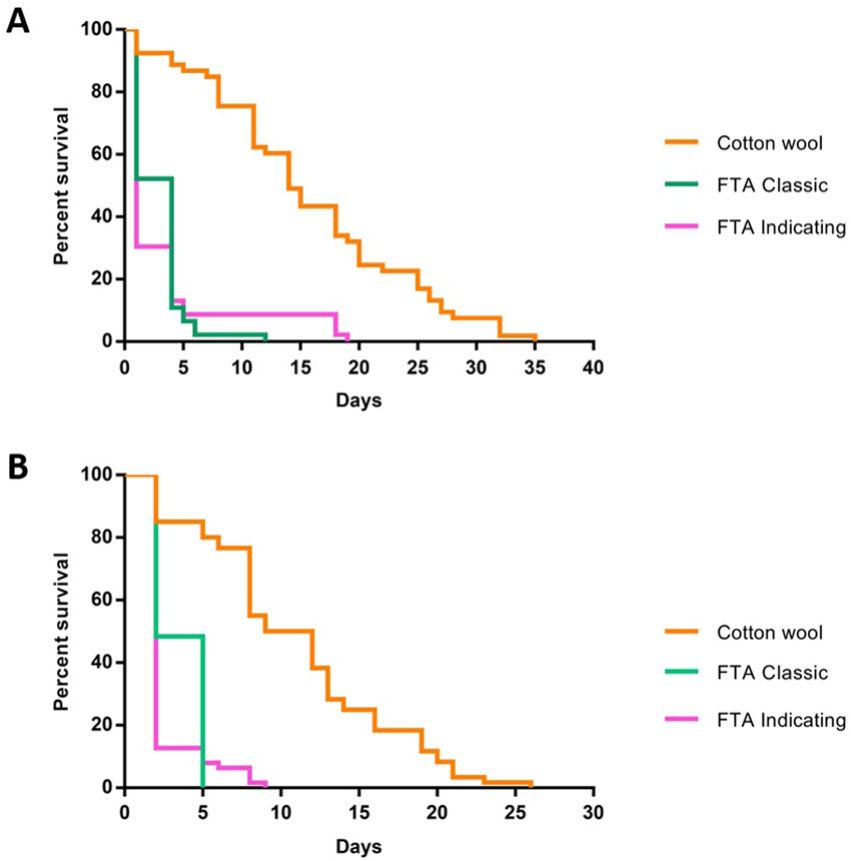
Kaplan-Meier survival curves for three groups of mosquitoes provided with glucose on cotton wool, classic FTA cards or indicating FTA cards. The survival distributions were significantly different for both mosquito species. (**A**) *An*. *stephensi* (Log-Rank statistic Χ^2^_df = 2_ = 82.88, *p* < 0.0001). (**B**) *An*. *coluzzii* (Log-Rank statistic Χ^2^_df = 2_ = 119.4, *p* < 0.0001).

We performed mosquito survival experiments using *An*. *stephensi* (SK strain) and *An*. *coluzzii* (N’gousso strain) to compare the survival rates of mosquitoes feeding on glucose from classic and indicating FTA cards (Supplementary Figure S1B) as compared with the standard cotton wool set up, to determine the effects of continual sugar feeding on these different substrates. Survival of both species of *Anopheles* mosquitoes provided with glucose on both types of FTA cards was significantly reduced compared to those given glucose on cotton wool (Fig. 5). The effect was significant for both *An*. *stephensi* (Log-Rank statistic Χ^2^_df = 2_ = 82.88, *p* < 0.0001) and *An*. *coluzzii* (Log-Rank statistic Χ^2^_df = 2_ = 119.4, *p* < 0.0001) suggesting mortality from sugar feeding on FTA cards is independent of Anopheline mosquito species. Furthermore, the greatest mortality occurs within the first few days of feeding on both classic and indicating FTA cards and by day 5 less than 10% of mosquitoes were alive compared to over 80% for those feeding on cotton wool. These results suggest a strong lethal effect of feeding on FTA cards on Anopheline mosquitoes, which could have a secondary benefit of being a toxic sugar source in field settings.

## Discussion

In this study, we have demonstrated that Anopheline mosquitoes expel malaria sporozoites while feeding on sugar sources under laboratory settings. Using qPCR techniques, we were able to detect the sporozoites of both *P*. *falciparum* and *P*. *berghei* from small groups of sugar-feeding mosquitoes from cotton wool and, with greater sensitivity, from Whatman FTA cards. Collectively, this demonstrates the potential of these methods to enhance investigations into mosquito-malaria interactions and malaria transmission in both the laboratory and field.

The ability to simply and rapidly detect sporozoites from a sugar-feeding substrate without sacrificing individual mosquitoes allows for a range of beneficial experimental applications. These include reduced cost and expert time, precluding the need for extensive training or laboratory equipment as is required for salivary gland dissections or ELISA-based approaches. Furthermore, the approach of using cotton wool which has no significant effects on mosquito mortality allows for more detailed investigations into the development of sporozoites within individual mosquitoes under variable laboratory conditions (e.g. ambient temperature or relative humidity). Such experiments are key to our understanding of the impact of environmental conditions on malaria transmission dynamics, yet the need to kill and dissect a mosquito to detect sporozoites has meant that assessing the progression of sporogony within an individual mosquito has not been possible, even with other rapid real-time PCR assays^23^. Furthermore, it is often thought that once a mosquito becomes infectious, it remains infectious (that is, it continues to expel sporozoites) for the rest of its life, which often does not extend much longer than the duration of sporogony. Here, we were able to detect *P*. *falciparum* sporozoites in cotton wool samples from 18 to 24 dpi but not from 26 dpi. This could therefore support previous observations showing that mosquitoes do not necessarily remain infectious for life and that the number of sporozoites in salivary glands can be depleted by repeated feeding^24^, due to physiological changes in salivary glands^15^ or can degenerate due to factors such as time and temperature^25,26^. However, further experiments involving sporozoite counts over time following sugar feeding would be required to draw definitive conclusions regarding this effect. Interestingly, another independent study recently published after completion of our experimental work also provides evidence that malaria sporozoites can be expelled on honey-coated FTA cards after only 12 days post infection using Taqman PCR analysis targeting the 18 S rRNA gene^27^.

The detection of malaria sporozoites on sugar-soaked FTA cards indicates the potential to use this methodology in malaria-endemic field settings. Firstly, FTA cards preserve both RNA and DNA without the need for a cold storage chain. FTA cards have been successfully used for detection of malaria gametocytes at sub-microscopic levels after the cards were first stored under hot and humid tropical conditions such as those encountered during sampling, followed by a storage period in variable conditions such as those during transportation^28^. When biological material comes into contact with the FTA card matrix, cells are lysed and nucleic acids are immobilised and stabilised within the matrix. This preserves both pathogen and mosquito DNA for either immediate processing or long-term storage, with no loss to PCR efficiency even when stored at room temperature^29^. To obtain DNA, FTA cards can be washed, leaving clean DNA to be extracted, and our optimised protocols for eluting DNA provide an ideal chain to move from field settings to PCR detection of malaria sporozoites in the laboratory.

Secondly, FTA cards inactivate pathogens such as malaria and arboviruses upon contact and prevent the growth of bacteria and other hazardous and contaminating microorganisms. Manuka honey can also be used as the sugar source to provide additional anti-bacterial protection^19,30^. This would facilitate the use of the cards for extended periods of time at high temperatures in tropical areas where malaria transmission occurs, whilst maximising safety during collection, transport and storage. The cards can be sent through regular shipping methods without the need for hazardous labelling, therefore providing an economical option of sample collection for malaria-endemic countries.

Thirdly, the application of this novel method of malaria transmission surveillance is particularly important when there is growing evidence that secondary Anopheline species are playing an important role in transmission^31^. In the field, live *Anopheles* mosquitoes are collected using several different methods such as human landing catches (HLC), exit trap collections, and CDC light traps^32^. Variations in the collection efficacy of each method and the time periods used can result in non-representative and poorly comparable samples and therefore skewed estimates of malaria transmission intensity. Following collection, large mosquito samples need to be sorted, identified and pooled prior to analysis, and only a fraction of these, often from presumed primary vectors, may be tested to estimate the prevalence of malaria in the mosquito population. This hurdle has similarly been seen in arbovirus surveillance, with an Australian study estimating that more than 47,000 mosquitoes would need to be collected and processed to obtain a single pool positive for Japanese Encephalitis virus^33^. FTA cards can simply be added to existing artificial traps with little or no modification to the design of the trap^34^. This would negate the need for extensive pre-sorting of samples and ensure malaria detection from any primary or secondary species that feeds on the card within the trap. Alternatively, trap-free bait stations containing FTA cards, similar to those set up for arbovirus surveillance^35,36^, could be set up in the field to allow mosquito feeding and subsequent detection of malaria. These FTA card approaches benefit from simplicity and preclude the need for extensive training of personnel; they therefore provide a simple, safe and cost-effective addition that could enhance existing surveillance regimes used by vector control programmes in remote areas. This may be particularly useful in elimination settings to detect the presence of sporozoites without extensive sampling and analysis of the mosquito population.

Finally, as FTA cards have already been used for detection of viruses in field settings^19,29,34,36–40^, sentinel-site surveillance for additional insect-borne pathogens could also theoretically be undertaken using FTA cards. Xenomonitoring of lymphatic filariasis and onchocerciasis (reviewed by Pilotte *et al*.^41^) has had limited programmatic application principally due to the difficulties in collecting mosquitoes and the costs of the molecular processing of large numbers of collected mosquitoes to determine parasite prevalence rates, which are often very low in field populations. For lymphatic filariasis, multiple mosquito genera play a role in transmission, so FTA cards could provide a simple tool to provide evidence of transmission without the need to screen large samples. FTA cards could also be used to detect pathogens with a postulated or confirmed mechanical mode of transmission from mosquitoes, such as *Francisella tularensis* subsp. *holarctica*^42,43^, or from other fly species including the house fly, *Musca domestica* (e.g. *Chlamydia trachomatis* or *Shigella* spp.)^44–47^.

## Methods

### Infection of mosquitoes with *Plasmodium*

*Anopheles stephensi* mosquitoes (SDA500 strain^48^) were infected with *P*. *berghei* (ANKA clone 507^49^) at Imperial College London (ICL) and London School of Hygiene and Tropical Medicine (LSHTM) or *Plasmodium falciparum* (NF54) (ICL) as described below.

Mosquitoes were infected with *P*. *berghei* by feeding on anaesthetised mice as described previously^50,51^. Briefly, *P*. *berghei* strain ANKA 507 was maintained in 4–10 week old female Tuck CD1 mice (Charles River) by serial blood passage (up to a maximum of eight passages). Hyper-reticulocytosis was induced 2–3 days before infection by treating mice with 200 µl intraperitoneal (i.p.) phenylhydrazine chloride (PHz; 6 mg.ml^−1^ in PBS; ProLabo, UK). Stock mice were infected by i.p. injection of blood containing parasites, and infections were monitored on Giemsa-stained tail blood smears. To generate infected mosquitoes, groups of five 4–10-week-old female PHz-treated CD1 mice were infected with *P*. *berghei* ANKA 2.34 by syringe inoculation (i.p.), followed by feeding to mosquitoes at days 3–4 post-infection. On days 3–4, five infected mice were anaesthetized and exposed to cages containing 500 starved female *An*. *stephensi* mosquitoes. Unfed mosquitoes were removed and fed ones were maintained on 8–10% (w/v) fructose, 0.05% (w/v) p-aminobenzoic acid and maintained at 19–20 °C and 80% relative humidity until required.

For *P*. *falciparum* infections, erythrocytic stages of the NF54 isolate were cultured as described previously^52^ followed by induction of gametocytogenesis^52^. Ten to 18-day old cultures demonstrating exflagellation of male gametocytes were then added to fresh human red blood cells (group A, National Blood Service, UK) with heat-inactivated human AB serum (National Blood Service, UK) at a packed cell volume of ∼40% and introduced into plastic membrane feeders. To generate infected mosquitoes, *An*. *stephensi* were allowed to feed on the membrane feeders for 25–30 min, and then maintained at 26 °C and 80% relative humidity until required.

### Collection of saliva from mosquitoes (‘forced salivation’)

*Plasmodium berghei*-infected female *An*. *stephensi* mosquitoes at 19 dpi were anesthetised using carbon dioxide and wings and legs were removed. The proboscis of each female was inserted into a pipette tip containing 100 μl of 10% glucose solution and was left to salivate for 2 hours (Fig. 1). Care was taken to check that the proboscis was continually in the glucose solution for the duration of the collection period.

### DNA extraction

DNA was extracted from individual whole mosquitoes using the DNeasy Blood and Tissue Kit (Qiagen, Manchester, UK) according to the manufacturer’s instructions. Modifications to this extraction protocol were made to extract genomic DNA from saliva, cotton wool samples and classic (white) and indicating (pink to white colour change upon sample addition) Whatman FTA cards. 100 µL of the 10% glucose solution containing saliva was added to 80 µL of ATL buffer to obtain the correct volume for the addition of 20 µL proteinase K. For cotton wool and FTA card sections, a working solution of 180 µL ATL buffer + 20 µL proteinase K + 200 μL AL buffer (total 400 µl) was added to these samples in 1.5 mL Eppendorf tubes and vortexed for 20 sec. Samples were incubated for 3 hours at 56 °C with vortexing every 1 hour. The remaining steps of the DNeasy Blood and Tissue Kit extraction protocol resulted in DNA eluted in 100 μL of AE buffer and stored at −20 °C prior to PCR analysis.

### Detection of *P*. *berghei*

Detection of *P*. *berghei* was undertaken using a real-time PCR targeting a 94-bp sequence of the *P*. *berghei* cytochrome b (*cytb*) mitochondrial gene (Accession No. DQ414645)^53^. The primers used for PCR were 5′-TGGGGACAAATGAGTTACTGG-3′ and 5′-CAGTGTATCCTCCACATAACCAA-3′. PCR reactions were prepared using a Quantinova SYBR green mix (Qiagen) as preliminary trials revealed this provided the most sensitive but specific detection method. Each reaction contained 5 µL of master mix, a final concentration of 1 µM of each primer, 1 µL of PCR grade water and 2 µL template DNA in a final reaction volume of 10 µL. Prepared reactions were run on a LightCycler® 96 System (Roche Diagnostics, West Sussex, UK) for 15 mins at 95 °C, followed by 40 cycles of 95 °C for 15 s and 60 °C for 1 min. Amplification was followed by a dissociation curve (95 °C for 10 s, 65 °C for 60 s and 97 °C for 1 s) to ensure the correct target sequence was being amplified. PCR results were analysed using LightCycler® 96 software (Roche Diagnostics).

In order to determine the assay’s limit of detection, we dissected the salivary glands from 10 *P*. *berghei*-infected mosquitoes and estimated the total number of sporozoites present for detection of DNA by counting using a haemocytometer. Pooled salivary glands were sequentially ten-fold diluted until 1:100,000 and the assay run with each dilution to determine the Ct threshold limit of detection.

### Detection of *P. falciparum*

Detection of *P*. *falciparum* was undertaken using a real-time PCR targeting a 120-bp sequence of the *P*. *falciparum* cytochrome c oxidase subunit 1 (*cox1*) mitochondrial gene^22,54^. Primers used for the PCR were 5′-TTACATCAGGAATGTTATTGC-3′ and 5′-ATATTGGATCTCCTGCAAAT-3′. This assay was selected due to sensitivity and specificity to detect mitochondria of all stages of *P*. *falciparum*. In addition, PCR reactions were prepared using FastStart SYBR Green Master mix (Roche Diagnostics) as preliminary trials revealed this provided the most sensitive but specific mastermix. Each reaction contained 5 µL of master mix, a final concentration of 1 µM of each primer, 1 µL of PCR grade water and 2 µL template DNA, to a final reaction volume of 10 µL. Prepared reactions were run on the LightCycler® 96 System (Roche Diagnostics) for 15 mins at 95 °C, followed by 35 cycles of 95 °C for 15 s and 58 °C for 30 s. Amplification was followed by a dissociation curve (95 °C for 10 s, 65 °C for 60 s and 97 °C for 1 s) to ensure the correct target sequence was being amplified. Positive controls from genomic DNA extracted from a cultured *P*. *falciparum*-infected blood (parasitaemia of ~10%) and the World Health Organization (WHO) International Standard for *P*. *falciparum* DNA^55^ were included on each run in addition to no template controls (NTCs). PCR results were analysed using the LightCycler® 96 software (Roche Diagnostics).

### Mosquito survival

Newly emerged *An*. *stephensi* (SK strain) and *An*. *coluzzii* (N’gousso strain^56^) mosquitoes were provided with 10% glucose and 0.05% para-aminobenzoic acid (PABA) mixture *ad libitum* on classic and indicating FTA cards and on cotton wool, and were kept at 26 °C and 70% relative humidity. Survival was monitored on a daily basis to determine the effects of continual sugar feeding on FTA cards.

### Statistical analysis

GraphPad Prism 7 was used to generate Box and whisker plots, Heat maps and Kaplan-Meier survival curves.

### Ethics

Animal work was conducted under UK Home Office license and approval in accordance with the United Kingdom Animals (Scientific Procedures) Act 1986 implementing European Directive 2010/63 for the protection of animals used for experimental purposes. All methods were carried out in accordance with relevant guidelines and regulations and approval was obtained from the LSHTM Animal Welfare Ethics Review Board and the ICL Animal Welfare and Ethical Review Body, with animal welfare assessed daily.

### Data availability

All data generated or analysed during this study are included in this published article (and its Supplementary Information files).

## Electronic supplementary material

Supplementary Figure S1
