# Supplementary material for: Detection of malaria sporozoites expelled during mosquito sugar feeding
Source: Sci Rep. 2018 May 15;8:7545. doi: 10.1038/s41598-018-26010-6 (PMC5954146; doi:10.1038/s41598-018-26010-6)
Supplement: Supplementary file 1 — Supplementary Figure S1 [file 41598_2018_26010_MOESM1_ESM.pdf]

Detection of malaria sporozoites expelled during mosquito sugar feeding

Brugman, V. A., Kristan, M., Gibbins, M. P., Angrisano, F., Sala, K.A., Dessens, J. T., Blagborough, A. M., & Walker, T.

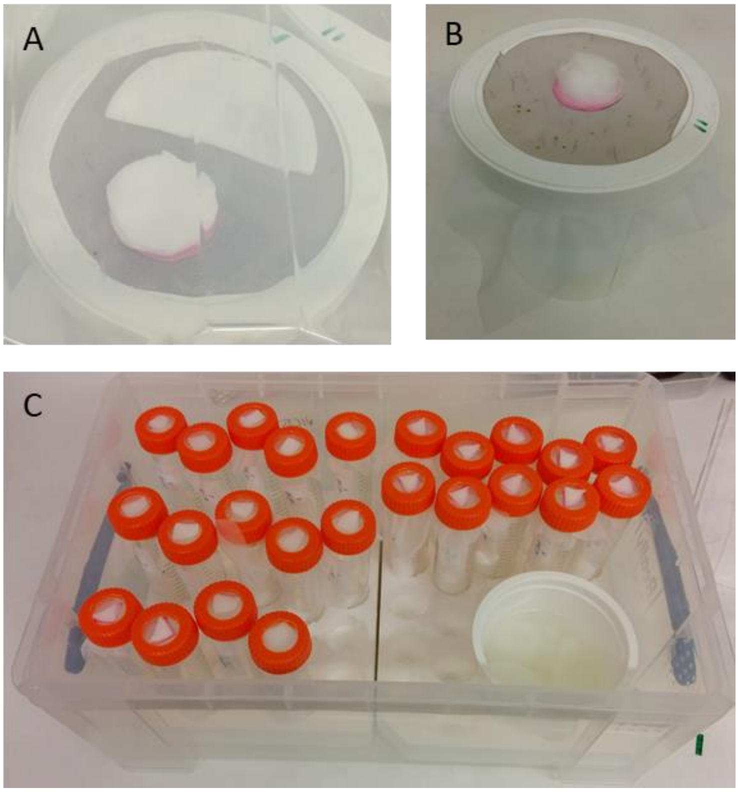

Supplementary Figure S1: Experimental setup for sugar-feeding *Plasmodium*-infected *Anopheles* mosquitoes. (A) Simultaneous supply of sugar-soaked indicating Whatman FTA card and sugar-soaked cotton wool pad; (B) Supply of sugar via indicating Whatman FTA card only; (C) Groups of three mosquitoes maintained in small tubes with access to sugar via either indicating Whatman FTA card or sugar-soaked cotton wool pad. Note that for the FTA card setups, sugar-soaked cotton wool was placed on top of each FTA card in order to prevent drying, but mosquitoes were only able to consume sugar from the cards.
